# Combining Adoptive Cell Therapy with Cytomegalovirus-Based Vaccine Is Protective against Solid Skin Tumors

**DOI:** 10.3389/fimmu.2017.01993

**Published:** 2018-01-16

**Authors:** Jeremy M. Grenier, Stephen T. Yeung, Zhijuan Qiu, Evan R. Jellison, Kamal M. Khanna

**Affiliations:** ^1^Department of Immunology, University of Connecticut Health, Farmington, CT, United States; ^2^Department of Molecular Genetics and Microbiology, Center for Infectious Diseases, Stony Brook University, Stony Brook, New York, NY, United States

**Keywords:** vaccine, checkpoint blockade, adoptive cell therapy, melanoma, cytomegalovirus

## Abstract

Despite many years of research, cancer vaccines have largely been ineffective in the treatment of established cancers. Many barriers to immune-mediated destruction of malignant cells exist, and these likely limit the efficacy of cancer vaccines. In this study, we sought to enhance the efficacy of a cytomegalovirus (CMV)-based vaccine targeting melanoma by combining vaccination with other forms of immunotherapy. Adoptive cell therapy in humans and in animal models has been shown to be effective for tumor regression. Thus, in this study, we assessed whether CMV-based vaccines in combination with adoptively transferred antitumor T cells could provide greater antitumor protection than either therapy alone. Our results show that adoptive cell therapy greatly enhanced the antitumor effects of CMV-based vaccines targeting the foreign model antigen, OVA, or the melanoma differentiation antigen, gp100. Combination adoptive cell therapy and vaccination induced the upregulation of the inhibitory ligands, PD-L1, and Qa-1^b^, on B16 tumor cells. This expression paralleled the infiltration of tumors by vaccine-stimulated T cells which also expressed high levels of the receptors PD-1 and NKG2A/C/E, suggesting a potential mechanism of tumor immune evasion. Surprisingly, therapeutic blockade of the PD-1/PD-L1 and NKG2A/Qa-1^b^ axes did not delay tumor growth following vaccination, suggesting that the presence of inhibitory ligands within malignant tissue may not be an effective biomarker for successful combination therapy with CMV-based vaccines. Overall, our studies show that therapeutic CMV-based vaccines in combination with adoptive T cell transfer alone are effective for tumor rejection.

## Introduction

After decades of research, immunotherapy has finally joined surgery, radiotherapy, and chemotherapy as a standard treatment modality in clinical oncology (1–3). Currently approved immunotherapies target inhibitory receptors on T cells. Unfortunately, not all patients respond to these inhibitors. Recent work has suggested that response is correlated with T cell infiltration and an “inflamed” tumor phenotype (4). Thus, methods for converting “non-inflamed” to “inflamed” tumors are needed to treat this subset of patients.

Vaccination is one potential method to generate an adaptive immune response against tumor cells and has been an active area of research for decades but yielding little clinical benefit as monotherapy (5, 6). Our lab has previously generated a novel vaccine for melanoma utilizing recombinant Cytomegalovirus (CMV) as a vaccine vector (7). Several characteristics make CMV an attractive vaccine vector: (1) CMV elicits a prolonged CD8^+^ T cell response characterized by the accumulation of high frequencies of virus-reactive T cells over the lifetime of the host (8). (2) In contrast to other persistent infections, CMV-specific T cells retain effector functions months after initial infection (9). (3) Prior CMV infection does not induce protective immunity, allowing for several rounds of vaccination using this vector. (4) The robust T cell response generated by CMV is also produced by attenuated, replication-deficient vectors (10). These properties have led several groups to test CMV vectors as vaccine platforms for infectious disease (11–13).

Our lab has previously hypothesized that a persistent vaccine-stimulating lifelong T cell responses against tumor antigen would be an effective cancer immunotherapy (7, 14). To test this, our group developed a vaccine based on murine cytomegalovirus (MCMV) targeting a shared melanoma antigen, gp100. By mutating gp100 within a CD8^+^ T cell epitope, we were able to generate gp100-reactive T cells which delayed metastatic tumor progression in the lungs following vaccination with MCMVgp100KGP. However, vaccinated mice eventually succumb to disease (7). In the current study, we tested whether MCMV-based vaccines could synergize with other established immunotherapies in a murine solid tumor model of melanoma. Adoptive cell therapy is a form of immunotherapy in which large numbers of tumor-reactive T cells are transferred into tumor-bearing mice or humans (15). Our previous study showed that MCMVgp100KGP induced modest frequencies of tumor-reactive T cells (7). This is likely due to low precursor frequency of T cells recognizing the unaltered gp100 epitope. We hypothesized that adoptive cell therapy could overcome this barrier and synergize with MCMV-based vaccines to produce more effective and prolonged antitumor responses. Importantly, the effectiveness of adoptive cell therapy also seems to correlate with persistence of transferred cells long term (16). Thus, in this study, we also tested if CMV-based vaccines could sustain adoptively transferred cells long term and result in augmented tumor regression. Our results show that a MCMV-based vaccine targeting a model neo-epitope is highly effective in treating melanoma expressing the same neo-epitope, and combining this vaccination with adoptive cell therapy completely cures mice of disease. Similarly, combining MCMVgp100KGP vaccination with adoptive cell therapy delays growth of established tumors. In both models, MCMV-based vaccination maintains adoptively transferred cells at low frequencies in peripheral blood. Following vaccination, tumors take on an “inflamed” phenotype characterized by T cell infiltration and expression of counter-regulatory molecules, such as PD-L1 and Qa-1^b^. However, the protection provided by MCMV-based vaccines and adoptive T cell therapy was as effective as combination immunotherapy targeting several counter-regulatory pathways. This suggests that vaccine-induced PD-L1 and Qa-1^b^ expression may not be the most accurate predictors of response to checkpoint blockade in some tumors in combination with viral-based tumor vaccines.

## Materials and Methods

### Mice

Female C57BL/6 mice (6–8 weeks old) were purchased from Charles River (Frederick, MD, USA). All mice used in these studies were between 6 and 12 weeks of age at the start of the experiment. Breeding pairs of PMEL mice (B6.Cg-Thy1a/Cy Tg(TcraTcrb)8Rest/J) were purchased from the Jackson Laboratory (Bar Harbor, ME, USA) and bred in house. OT-I/RAG^−/−^ mice were provided by Dr. Leo Lefrançois (University of Connecticut Health Center). Mice were housed at the University of Connecticut Health Center in a pathogen-free facility, and all experiments were performed with approval by the University of Connecticut Health Center Institutional Animal Care and Use Committee.

### Cell Lines and Viruses

B16F10 and B16ova were provided by Dr. Leo Lefrançois (University of Connecticut Health Center). B16F10-RFP was purchased from AntiCancer, Inc. (San Diego, CA, USA). B16F10 cells were cultured in DMEM media (Life Technologies) supplemented with 10% fetal bovine serum (Life Technologies), 1 mM sodium pyruvate (Life Technologies), 1% non-essential amino acids (Life Technologies), and 1% Penicillin/Streptomycin (Life Technologies). B16ova cells were cultured in B16F10 culture media supplemented with 500 µg/mL G418 (Life Technologies). B16F10-RFP were cultured according to supplier’s instructions in RPMI-1640 media (Life Technologies) supplemented with 10% fetal bovine serum, 2 mM l-glutamine (Life Technologies), 1% Penicillin/Streptomycin, and 400 µg/mL G418. Mouse embryonic fibroblasts were cultured in DMEM media supplemented with 5% fetal bovine serum, 1% non-essential amino acids, 1% sodium pyruvate, 2 mM l-glutamine, and 1% Penicillin/Streptomycin. M2-10B4 (a kind gift from Dr. Christopher Snyder; Thomas Jefferson University) were cultured in RPMI-1640 media supplemented with 10% fetal bovine serum, 2 mM l-glutamine, and 1% Penicillin/Streptomycin.

Murine cytomegalovirus-OVA was provided by Dr. Carol Wu (University of Connecticut Health Center). Wild–type (WT) MCMV, MCMVgp100, and MCMVgp100KGP were previously generated in the lab using the MCMV BAC pSM3fr-MCK-2fl provided by Dr. Barbara Adler (Ludwig-Maximilians-University Munich, Germany) (7). Briefly, the full-length murine gp100 coding sequence or the altered sequence (gp100KGP) was inserted into the MCMV ie2 locus under the control of the HCMV ie1 promoter (7). Previously generated viruses (WT MCMV, MCMVgp100, and MCMVgp100KGP) were expanded using murine embryonic fibroblasts or the bone marrow stromal cell line, M2-10B4, as previously described (17).

### Tumor Challenge Experiments

Female C57BL/6 mice received an intradermal injection of 10^5^ B16F10 or B16F10 expressing RFP (B16RFP) or 3 × 10^5^ B16ova. Mice were then vaccinated *via* i.p. injection with 10^5^ PFU WT or recombinant MCMV on Day 5 or Day 8, depending on the experiment. In some experiments, splenocytes corresponding to 10^5^ CD8^+^ PMEL cells from naïve PMEL mice or 10^5^ CD8^+^ OT-I cells from OT-I/Rag^−/−^ mice were transferred into naïve WT tumor-bearing mice 2 h prior to vaccination with WT or recombinant MCMV. Intradermal tumor growth was monitored every 2–3 days by measuring length and width of the tumor using calipers and multiplying to calculate surface area. Mice were euthanized when tumors reached >100 mm^2^ or ulcerated. In some experiments, tumor-bearing mice also received i.p. injections of anti-PD-1 antibody (RMP1-14; BioXcell), anti-Qa-1^b^ (4C2.4A7.5H11; BioXcell), or isotype controls.

### Flow Cytometry

Tumor tissue was mechanically dissociated and digested in 0.7 mg/mL Collagenase D (Roche) and 3 mg/mL DNase I (Roche) for 30–45 min to obtain single-cell suspension. For experiments looking at tumor-infiltrating leukocytes (TIL), TIL were isolated using Percoll gradient prior to staining. For experiments looking at B16RFP^+^ tumor cells, cells were stained immediately after digestion for 20 min. Cells were blocked with anti-CD16/32 (clone 93; Biolegend) prior to surface staining. Antibodies against the following antigens were used: CD11a (clone M17/4; ThermoFisher), CD8a (clone 53-6.7; BD Biosciences or Biolegend or eBioscience), CD45 (clone 30-F11; Invitrogen or eBioscience), CD45.1 (clone A20; Biolegend), CD3 (clone eBio500A2; eBioscience), CD45.2 (clone 104; eBioscience), CD90.1 (clone OX-7; BD Biosciences), CD127 (clone A7R34; eBioscience or clone SB/199; Biolegend), KLRG1 (clone 2F1/KLRG1; Biolegend or eBioscience), PD-1 (clone RMP1-30; eBioscience), NKG2A/C/E (clone 20d5; eBioscience), LAG3 (clone C9B7W; Biolegend), CD44 (clone IM7; eBioscience or Biolegend or BD Biosciences), Ly6C (clone HK1.4; Biolegend), Ly6G (clone 1A8; Biolegend), CD11b (M1/70; eBioscience or Biolegend), Qa-1^b^ (clone 6A8.6F10.1A6; Miltenyi Biotec), and PD-L1 (10F.9G2; Biolegend).

### Statistical Analysis

Statistical tests were performed in Prism (Graphpad). For tumor growth experiments, tumor growth curves were compared using two-way repeated measures ANOVA. For other experiments, a student *t*-test was used when comparing two groups, and a one-way ANOVA was used when comparing more than two groups. A paired *t*-test was used to compare inhibitory receptor expression in blood and TIL from the same mouse. Survival curves were analyzed using Log-rank test in Prism.

## Results

### MCMV-OVA Maintains Adoptively Transferred Antitumor T Cells at Low Frequency Long Term

**Figure 1:**
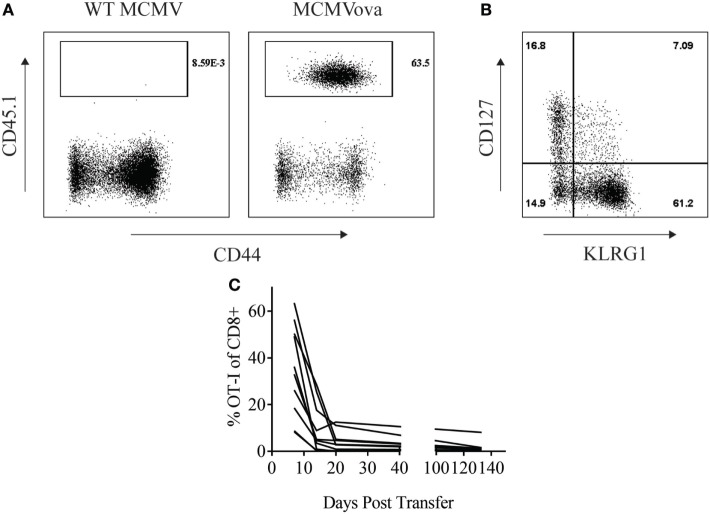
Murine cytomegalovirus (MCMV)-OVA maintains adoptively transferred OT-I cells at low frequency long-term. Wild-type (WT) CD45.2^+^ mice received 10^5^ CD45.1^+^ OT-I cells i.v. followed by i.p. vaccination with 10^5^ WT MCMV or MCMV-OVA. Peripheral blood was collected at various time points after transfer and processed for flow cytometry. **(A)** Representative plots from peripheral blood of mice receiving OT-I transfer and vaccination with WT MCMV or MCMV-OVA 7 days prior. Gated on CD8^+^ cells. **(B)** Representative plot showing phenotype of OT-I cells from mice vaccinated with MCMV-OVA 7 days prior. Gated on CD8^+^CD45.1^+^ cells. **(C)** Frequency of CD45.1^+^ of CD8^+^ cells over time in peripheral blood of mice vaccinated with MCMV-OVA. Each curve is representative of one mouse. Data are accumulated from two independent experiments with 10 mice/group total.

Adoptive cell therapy is a form of cancer immunotherapy that involves infusing large numbers of *ex vivo*-stimulated tumor-specific T cells into patients (15). Persistence of transferred cells over time correlates with improved clinical responses following adoptive cell therapy (16). Several groups have tried to utilize the persistent nature of CMV infection to enhance this immunotherapy by redirecting CMV-specific T cells to target tumor antigen (18, 19). Similarly, we wondered if CMV-based vaccines could be used to enhance the persistence of adoptively transferred cells. To test this, 10^5^ OT-I CD8^+^ T cells were transferred into naïve mice that were then vaccinated with either 10^5^ PFU WT MCMV or MCMV-OVA. As suggested by previous literature, MCMV-OVA vaccination stimulated a potent expansion of transferred T cells which was not seen following vaccination with WT MCMV (Figure 1A). Previous studies in adoptive cell therapy have shown that cells with a memory phenotype persist longer in recipients (20). We, therefore, assessed the phenotype of transferred OT-I cells following MCMV-OVA vaccination. Similar to previous studies, stimulated OT-I cells displayed an “effector memory” phenotype (KLRG1^+^CD127^lo^) in peripheral blood suggestive of a short-lived cell population (Figure 1B). Despite this same phenotype, MCMV inflationary T cell populations continue to accumulate over time due to continuous expansion of a small number of memory cells (9, 21). We, therefore, asked if transferred OT-I cells stimulated by MCMV-OVA persisted long term. To test this, mice were treated as in Figure 1A and OT-I frequency was followed over time in peripheral blood of mice. The majority of mice showed a progressive decline in OT-I frequency over time. Four months after transfer, OT-I cells were still detectable (<2% of CD8^+^) in blood in the majority of mice tested, and 1 of 10 mice showed a significant frequency of OT-I cells (8% of CD8^+^) 4 months after transfer (Figure 1C). While OT-I cells were still detectable in the majority of vaccinated mice several months after transfer, the frequency of transferred cells is much lower than we had hypothesized or had been shown in a previous study using recombinant MCMV (22).

### Adoptive Cell Transfer Enhances Efficacy of MCMV-OVA in Treatment of B16ova

**Figure 2:**
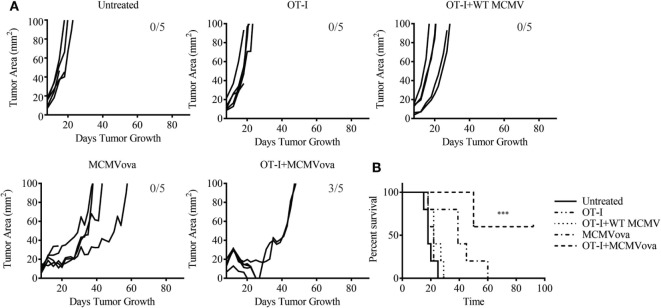
Adoptive cell therapy and murine cytomegalovirus (MCMV)-OVA vaccination cures mice of B16ova. Wild-type (WT) mice were challenged with B16ova i.d. and 8 days later were left untreated, treated with 10^5^ OT-I cells i.v., treated with 10^5^ PFU MCMV-OVA i.p., treated with OT-I transfer i.v. and 10^5^ PFU WT MCMV i.p., or treated with OT-I transfer i.v. and MCMV-OVA i.p. **(A)** Tumor growth curves of individual mice treated as shown. **(B)** Survival curves for experiment shown in **(A)**. *n* = 5 mice. Number of tumor-free mice at end of experiment shown in **(A)**. Data are representative of three independent experiments. ***p < 0.001.

Despite the low frequency of transferred cells at late time points, we were still curious if combination adoptive cell therapy and MCMV vaccination could impact solid tumor growth of a highly aggressive melanoma tumor. To test if adoptive cell therapy and MCMV-OVA could control disease in a solid tumor model, mice were inoculated with 3 × 10^5^ B16ova on the rear flank. Eight days later when tumors were visible, mice were injected with 10^5^ OT-I cells followed by 10^5^ PFU WT MCMV or MCMV-OVA. As seen in our previous study, mice receiving MCMV-OVA alone showed a delay in tumor growth and prolonged survival, yet ultimately succumbed to disease. By contrast, three of five mice receiving OT-I transfer and MCMV-OVA vaccination completely cleared their tumors and remained tumor-free for the remainder of the experiment (Figure 2). Thus, despite the low persistence of transferred cells in vaccinated mice, combination adoptive cell therapy and recombinant MCMV vaccination were able to cure mice of tumors expressing a foreign antigen.

### MCMVgp100KGP Stimulates Adoptively Transferred PMEL Cells but Maintains Them at Low Frequency Long Term

**Figure 3:**
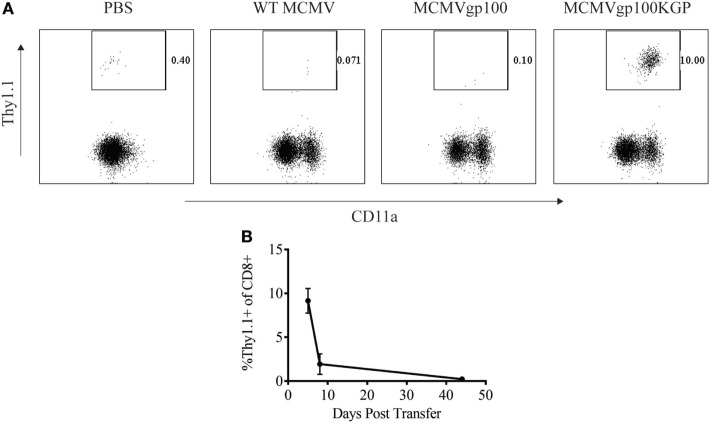
MCMVgp100KGP maintains adoptively transferred PMEL cells at low frequency long-term. Wild-type (WT) Thy1.2^+^ mice received 10^5^ Thy1.1^+^ PMEL cells i.v. followed by mock vaccination with PBS or vaccination with 10^5^ PFU WT murine cytomegalovirus (MCMV), MCMVgp100, or MCMVgp100KGP i.p. Peripheral blood was collected at various time points after transfer and processed for flow cytometry. **(A)** Representative plots from peripheral blood of mice receiving PMEL transfer and vaccination 5 days prior. Gated on CD8^+^ cells. **(B)** Frequency of Thy1.1^+^ of CD8^+^ cells over time in peripheral blood of mice vaccinated with MCMVgp100KGP. *n* = 4–5 mice/group. Data are representative of two independent experiments.

We next sought to determine how a CMV-based vaccine targeting an endogenous tumor antigen, gp100, would maintain antitumor T cell responses. Our lab has previously developed a recombinant MCMV expressing the modified melanoma antigen, gp100KGP (7). This virus expresses murine gp100 containing a mutation within a known CD8^+^ T cell epitope, which generates an endogenous T cell response to the native peptide. We first wanted to determine if MCMVgp100KGP could stimulate CD8^+^ PMEL cells which express a transgenic T cell receptor that recognizes the same epitope within gp100. To test this, naïve WT mice were injected with 10^5^ CD8^+^ PMEL cells followed by vaccination with WT MCMV, MCMV expressing native gp100, or MCMVgp100KGP. Five days following transfer, PMEL cells were detected at high frequency only in the blood of mice vaccinated with MCMVgp100KGP (Figure 3A), while recombinant virus expressing native gp100 did not stimulate transferred PMEL cells. We next asked if MCMVgp100KGP could promote the persistence of adoptively transferred PMEL cells. Similar to our findings with MCMV-OVA, MCMVgp100KGP stimulated a large expansion of adoptive transferred cells early but did not maintain transferred cells at high frequency in the blood long term (Figure 3B). Thus, in two models, MCMV-based vaccines maintain adoptively transferred cells in peripheral blood at low frequency long-term.

### Adoptive Cell Therapy and MCMVgp100KGP Vaccination Delays Solid Tumor Growth Therapeutically

**Figure 4:**
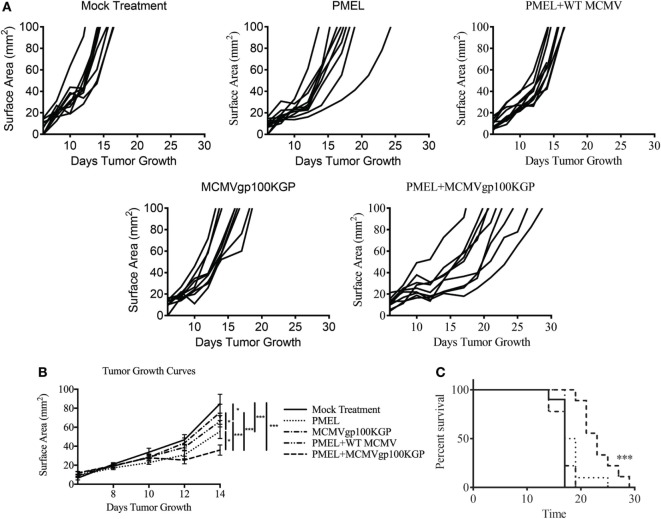
Adoptive cell therapy and MCMVgp100KGP vaccination delays B16F10 tumor growth. Wild-type (WT) mice were challenged with B16F10 i.d. and 5 days later were mock treated, treated with PMEL transfer, treated with PMEL transfer i.v. and 10^5^ PFU WT MCMV i.p., treated with 10^5^ PFU MCMVgp100KGP i.p. alone, or treated with PMEL transfer i.v. and 10^5^ PFU MCMVgp100KGP i.p. **(A)** Tumor growth curves of individual mice treated as shown. **(B)** Combined tumor growth curves and **(C)** survival from experiment shown in **(A)**. Data are accumulated from two independent experiments with *n* = 9–10 mice/group total. One mouse from the PMEL and MCMVgp100KGP group did not grow a tumor and was excluded. *p < 0.05; ***p < 0.001.

Given the encouraging results seen with adoptive transfer and MCMV-OVA vaccination, we next asked if this same combination therapy could treat tumors when targeting a native tumor antigen. To test this, mice were inoculated with 10^5^ B16F10, and 5 days later, mice received 10^5^ CD8^+^ PMEL cells followed by vaccination with WT MCMV or MCMVgp100KGP. As expected, mock vaccinated tumors grew unopposed with most mice succumbing to disease within 2 weeks (Figure 4). PMEL transfer alone, MCMVgp100KGP vaccination alone, or PMEL transfer followed by WT MCMV vaccination also had little effect on tumor growth. However, PMEL transfer followed by MCMVgp100KGP vaccination significantly delayed tumor growth and prolonged survival of tumor-bearing mice. This shows that increasing the precursor frequency of antitumor T cells through adoptive transfer can greatly enhance the clinical efficacy of MCMV-based vaccines in an antigen-specific manner.

### MCMVgp100KGP-Stimulated T Cells Express Inhibitory Receptors in the Tumor Microenvironment and Induce Expression of Immunosuppressive Pathways in Tumors

**Figure 5:**
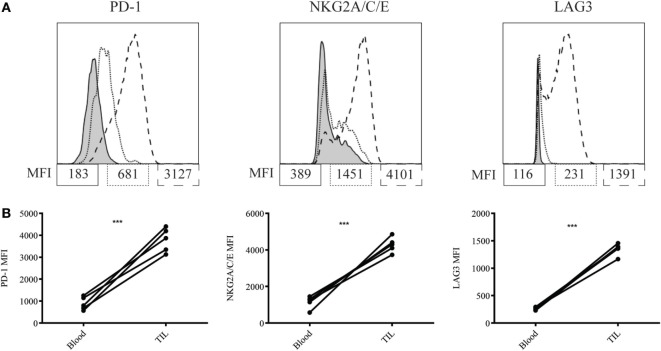
MCMVgp100KGP stimulated PMEL cell upregulate inhibitory receptors within the tumor microenvironment. Mice bearing B16F10 tumors were treated with PMEL transfer i.v. and 10^5^ PFU MCMVgp100KGP vaccination i.p. Six days later, peripheral blood and tumor tissue were harvested for flow cytometry. **(A)** Representative plots of inhibitory receptor expression on PMEL cells from peripheral blood (dotted lines) and tumor tissue (dashed lines). Shaded curves represent FMO controls. **(B)** Inhibitory receptor MFI on PMEL cells from peripheral blood vs. tumor tissue. ***p < 0.001. Paired *t*-test. *n* = 5 mice/group. Data are representative of three independent experiments.

**Figure 6:**
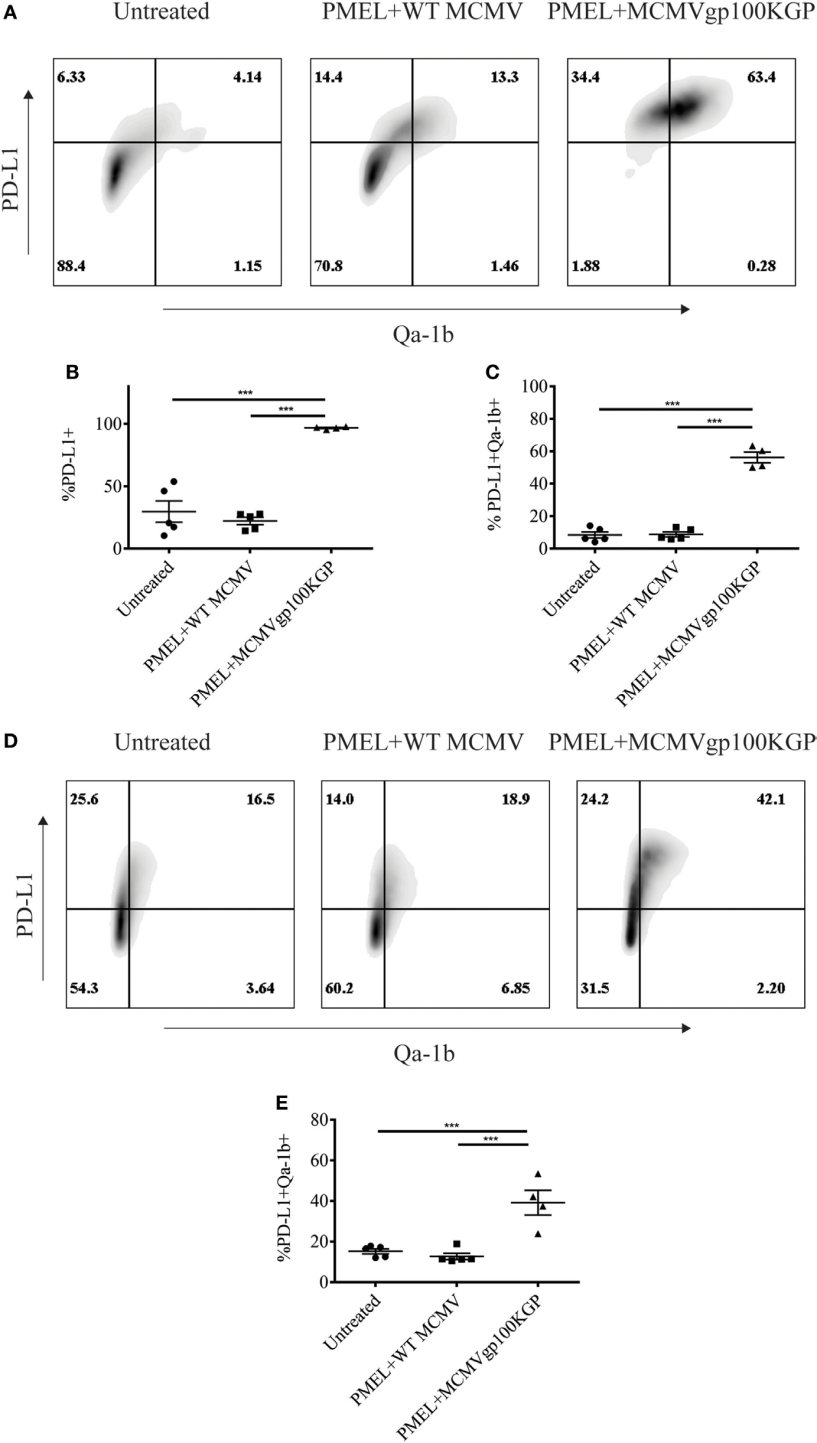
MCMVgp100KGP vaccination induces expression of PD-L1 and Qa-1^b^ within B16 tumors. Mice were challenged with B16F10 expressing RFP (B16RFP) i.d. and 5 days later left untreated, treated with PMEL transfer i.v. and 10^5^ PFU wild-type (WT) MCMV i.p., or treated with PMEL transfer i.v. and 10^5^ PFU MCMVgp100KGP i.p. Six days later, tumor tissue was harvested for flow cytometry. **(A)** Representative plots of RFP^+^ tumor cells. **(B)** Percentage of RFP^+^ cells expressing PD-L1. **(C)** Percentage of RFP^+^ cells expressing PD-L1 and Qa-1^b^. **(D)** Representative plots of CD45^+^CD8^−^ TIL. **(E)** Percentage of CD45^+^CD8^−^ TIL expressing PD-L1 and Qa-1^b^. ***p < 0.001; *n* = 4–5 mice/group. Data are representative of three independent experiments.

**Figure 7:**
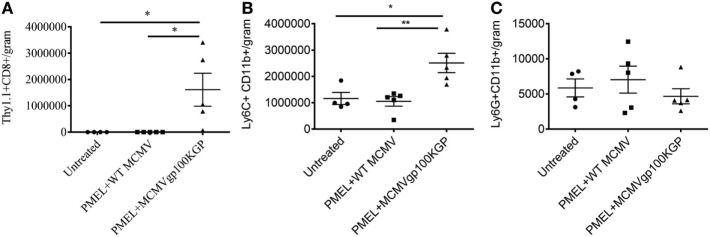
MCMVgp100KGP vaccination induces accumulation of Ly6C^+^ myeloid cells. Mice bearing B16F10 tumors were treated left untreated, treated with PMEL transfer i.v. and 10^5^ PFU wild-type (WT) MCMV i.p., or treated with PMEL transfer i.v. and 10^5^ PFU MCMVgp100KGP i.p. Six days later, tumor tissue was harvested for flow cytometry. Graphs show cell counts per gram tumor tissue for **(A)** Thy1.1^+^CD8^+^PMEL cells, **(B)** Ly6C^+^CD11b^+^ cells, and **(C)** Ly6G^+^CD11b^+^ cells. *p < 0.05; **p < 0.01; *n* = 4–5 mice/group. Data are representative of two independent experiments.

We next asked why our combination therapy did not completely clear tumors. To this end, we analyzed the phenotype of TIL for clues. Six days following vaccination with MCMVgp100KGP, PMEL cells within the tumor microenvironment expressed high levels of the inhibitory receptors LAG3, PD-1, and NKG2A compared to PMEL cells in the blood (Figure 5). Previous studies have suggested that expression of multiple inhibitory receptors correlates with cell exhaustion (23). PD-1 has two known ligands, PD-L1 and PD-L2, whose expression are regulated by IFNγ. NKG2A is an inhibitory receptor found on activated NK cells and some CD8^+^ T cells, and it recognizes the murine homolog of HLA-E, Qa-1^b^, whose expression is also regulated by IFNγ. Given the high expression of these inhibitory receptors on the surface of T cells within the tumor tissue, we next asked if the corresponding ligands were expressed within the tumor microenvironment. To test this, mice were inoculated with 10^5^ B16RFP and 5 days later were left untreated or given PMEL cells followed by vaccination with WT MCMV or MCMVgp100KGP. Six days following treatment, tumor tissue was excised and processed for flow cytometry. B16RFP cells recovered from untreated or control treated tumors showed little PD-L1 or Qa-1^b^ expression (Figures 6A–C). Strikingly, virtually all RFP^+^ tumor cells isolated from mice treated with MCMVgp100KGP immunotherapy were PD-L1^+^ and a significant percentage stained positive for both PD-L1 and Qa-1^b^ on the cell surface (Figures 6A–C). PD-L1 and Qa-1^b^ were also highly upregulated on CD45^+^ CD8^−^ immune cells within the tumor following MCMVgp100KGP immunotherapy (Figures 6D,E). In addition to these inhibitory molecules, we also noted a substantial increase in total number of Ly6C^+^ myeloid-derived suppressor cells within tumors following immunotherapy (Figure 7). These data show that immunotherapy-induced T cells rapidly induce the expression of several immunosuppressive pathways within the tumor microenvironment.

### PD-1 and Qa-1^b^ Blockade Does Not Improve Vaccination Response

**Figure 8:**
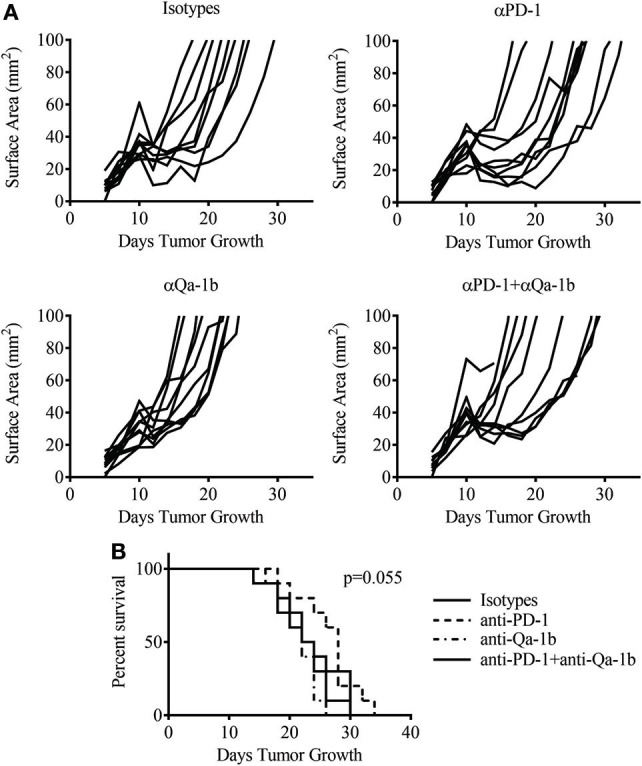
Dual checkpoint blockade does not enhance efficacy of MCMVgp100KGP vaccination. Mice were challenged with B16F10 i.d. and 5 days later received PMEL transfer i.v. and 10^5^ PFU MCMVgp100KGP vaccination i.p. Mice were treated with isotype, anti-PD-1, and/or anti-Qa-1^b^ antibodies i.p. on days 10, 12, and 14. **(A)** Tumor growth curves from individual mice. **(B)** Survival curves from different treatment groups. Data are accumulated from two independent experiments with 10 mice/group total.

PD-1 blockade is now a well-established therapy for metastatic melanoma and other cancers, while blocking antibodies to NKG2A are currently under development for cancer therapy. Given the high expression of these inhibitory pathways following therapy, we asked whether blockade of the PD-1/PD-L1 and NKG2A/Qa-1^b^ axes could improve the antitumor response generated by MCMVgp100KGP vaccination. To this end, tumor-bearing mice received 10^5^ PMEL cells and MCMVgp100KGP vaccination 5 days after tumor inoculation, followed by blocking antibodies to PD-1 and Qa-1^b^ or isotype controls on days 10, 12, and 14. Surprisingly, despite the high expression of PD-1/PD-L1 and NKG2A/Qa-1^b^ within the tumor, blockade of these pathways had no effect on tumor growth or survival in vaccinated mice (Figure 8). This suggests that the presence of these molecules may not be effective biomarkers for response to checkpoint inhibition. Other mechanisms of resistance may also be responsible for tumor progression.

## Discussion

In this study, we extended our previous observations testing a novel recombinant CMV vaccine for melanoma. Our previous work had shown that a recombinant CMV significantly delays disease progression in a metastatic model of melanoma (7). In the current study, we sought to enhance the efficacy of vaccination by combining CMV-based vaccines with other forms of immunotherapy in a solid skin tumor model.

Adoptive cell therapy is a form of immunotherapy involving the transfer of high frequencies of *ex vivo*-stimulated antitumor T cells into patients (15). A prominent study in this field showed that clinical response correlated with persistence of transferred cells in patients (16). Several groups have engineered CMV-specific T cells to express receptors for tumor antigens with the hope of improving cell persistence through viral stimulation of these cells (18, 19, 24). We, therefore, sought to ask a similar question: can a CMV-based vaccine sustain adoptively transferred antitumor T cells? Our results suggest that these cells can be maintained long-term albeit at a very low frequency. Our data stand in contrast to a recent paper by Turula et al. which showed that MCMV expressing ova could stimulate adoptively transferred OT-I cells at high frequency (22). Our conflicting results may be attributed to the different viruses used in our two studies or may be influenced by the number of cells transferred prior to vaccination. Turula et al. transfer 600 OT-I cells prior to vaccination, while in our study, mice received 10^5^ OT-I cells. This large frequency of transferred cells may reduce viral load and limit antigenic stimulation. Nonetheless, our data show that adoptive cell therapy greatly enhances the efficacy of CMV-based vaccines. Despite the low persistence of transferred OT-Is, OT-I transfer and MCMV-OVA vaccination completely cured mice of B16ova tumors. Similarly, PMEL transfer and MCMVgp100KGP vaccination significantly delayed growth of B16F10 tumors and increased survival of mice bearing established tumors.

Our study also highlights several immunosuppressive pathways that are upregulated in response to vaccination. PD-L1 is a notable T cell inhibitory ligand that is the target of several clinical immunotherapies. Clinical response to PD-1 blockade is associated with an “inflamed” tumor phenotype characterized by high levels of T cell infiltration and PD-L1 expression (25–28). B16F10 has previously been classified as a “non-immunogenic” tumor based on the resistance to immunotherapy (29). In this study, untreated B16F10 tumors express little PD-L1. Vaccination and infiltration by antitumor T cells significantly increases the expression of PD-L1, suggesting that vaccination can transform B16 tumors into “inflamed” tumors. We also noted the expression of several other immunosuppressive pathways, including NKG2A and its ligand Qa-1^b^. Qa-1^b^ is the murine homolog of HLA-E which has also been shown to protect malignant cells from T cell cytotoxicity (30, 31). Surprisingly, increased T cell infiltration and expression of these pathways following vaccination did not enhance the responsiveness of B16 tumors to blocking antibodies. These data highlight the difficulty in using the expression of T cell inhibitory molecules within the tumor microenvironment as markers for response to blocking antibodies. Instead, our data suggest that these molecules can be used as markers for successful vaccination.

Other pathways likely limit the effectiveness of vaccination in this tumor model. Herein, we also showed that vaccination induced the accumulation of Ly6C^+^ myeloid cells. Given that a previous study using PMEL transfer noted a similar accumulation and showed that these cells were indeed suppressive, we hypothesize that the Ly6C^+^ myeloid cells that accumulate in response to vaccination are also suppressive (32). This response may be one of the mechanisms limiting the efficacy of vaccination in our study. Future work will attempt to target these myeloid cells in combination with vaccination.

In summary, in this study, we attempted to enhance the efficacy of melanoma vaccines using CMV-based vectors. Adoptive cell therapy greatly enhanced the antitumor response to vaccination targeting both foreign and self-antigens. When targeting a foreign antigen, adoptive cell therapy and MCMV-based vaccination cured several mice of an aggressive solid tumor, suggesting that MCMV-based vaccines against neoantigens may have dramatic clinical results. Not surprisingly, this same combination targeting a self-antigen has a less dramatic clinical response. Vaccination induced several counter-regulatory mechanisms that were not overcome by blocking antibodies, suggesting that other tumor evasion mechanisms are at play. Overall, this study extends our earlier work showing that CMV-based vaccines are effective therapies against immunosuppressive solid tumors. Future work will determine which counter-regulatory responses are limiting vaccine efficacy in this tumor model. Nevertheless, the protection provided by CMV-based vaccines is more effective than checkpoint blockade alone in the B16 model of melanoma.

## Ethics Statement

The study was carried out in accordance with the University of Connecticut Health Center Institutional Animal Care and Use Committee. The protocol was approved by the University of Connecticut Health Center Institutional Animal Care and Use Committee.

## Author Contributions

JG and KK designed the study. JG and SY performed the experiments. ZQ and EJ provided reagents and analytic tools. JG, SY, and KK analyzed the data and wrote the paper.

## Conflict of Interest Statement

The authors declare that the research was conducted in the absence of any commercial or financial relationships that could be construed as a potential conflict of interest.
